# Cultivation and application of nicotine-degrading bacteria and environmental functioning in tobacco planting soil

**DOI:** 10.1186/s40643-023-00630-x

**Published:** 2023-02-01

**Authors:** Yiting Wang, Xiangyan Luo, Peng Chu, Heli Shi, Rui Wang, Jiale Li, Shixue Zheng

**Affiliations:** 1 State Key Laboratory of Agricultural Microbiology, College of Life Science and Technology, Huazhong Agricultural University, Wuhan, 430070 People’s Republic of China; 2 Enshi Branch, Hubei Tobacco Company, Enshi, 445000 Hubei People’s Republic of China

**Keywords:** Nicotine-degrading bacteria (NDB), Isolation, Metabolic pathway, Metagenomics, Compost of tobacco waste

## Abstract

Nicotine, a toxic and addictive alkaloid from tobacco, is an environmental pollutant. However, nicotine-degrading bacteria (NDB) and their function in tobacco planting soil are not fully understood. First, 52 NDB strains belonging to seven genera were isolated from tobacco soil. The most dominant genera were *Flavobacterium* (36.5%), *Pseudomonas* (30.8%), and *Arthrobacter* (15.4%), and *Chitinophaga* and *Flavobacterium* have not been previously reported. Then, two efficient NDB strains, *Arthrobacter nitrophenolicus* ND6 and *Stenotrophomonas geniculata* ND16, were screened and inoculated in the compost fertilizer from tobacco waste. The nicotine concentrations were reduced from 1.5 mg/g (DW) to below the safety threshold of 0.5 mg/g. Furthermore, strain ND6 followed the pyridine pathway of nicotine degradation, but the degrading pathway in strain ND16 could not be determined according to genomic analysis and color change. Finally, the abundance of nicotine-degrading genes in tobacco rhizosphere soil was investigated via metagenomic analysis. Five key genes, *ndhA*, *nctB*, *kdhL*, *nboR*, and *dhponh*, represent the whole process of nicotine degradation, and their abundance positively correlated with soil nicotine concentrations (*p* < 0.05). In conclusion, various NDB including unknown species live in tobacco soil and degrade nicotine efficiently. Some key nicotine-degrading genes could be used in monitoring nicotine degradation in the environment. The fermentation of compost from tobacco waste is a promising application of efficient NDB.

**Graphical Abstract:**

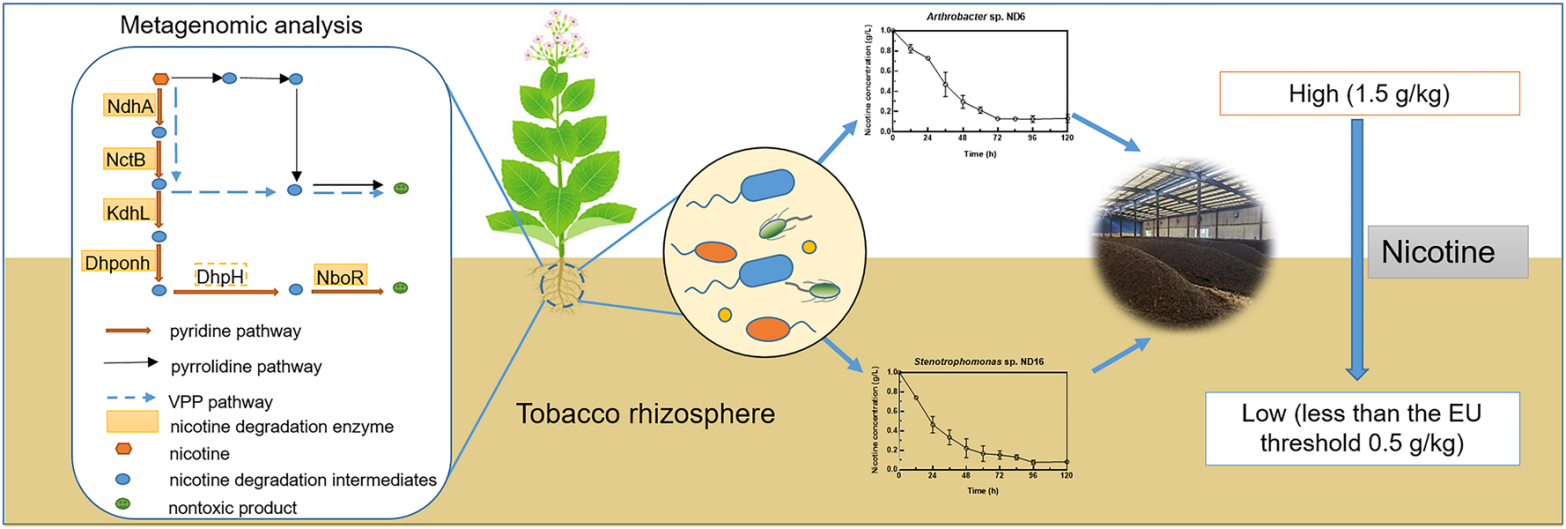

**Supplementary Information:**

The online version contains supplementary material available at 10.1186/s40643-023-00630-x.

## Highlights

- *Chitinophaga* spp. and *Flavobacterium* spp. were first confirmed as NDB.
- 52 NDB strains from seven genera were isolated from tobacco rhizosphere soil.
- Strains ND6 and ND16 efficiently degraded nicotine in medium and tobacco waste compost.
- ND16 may have a new nicotine-degrading pathway by phenotype and genome analysis.
- Abundance of five nicotine-degrading genes in soil correlated well with nicotine concentrations.

**Supplementary Information:**

The online version contains supplementary material available at 10.1186/s40643-023-00630-x.

## Introduction

Nicotine is widely found in *Solanaceae* plants and is abundant in tobacco, accounting for 2–8% of its dry weight. The chemical molecules of nicotine are mainly heterocyclic compounds composed of pyridine and pyrrole rings. Nicotine is not only toxic and carcinogenic to humans but also poses a great threat to the environment, resulting in a serious damage to the ecological balance in soil and groundwater (McGrath-Morrow et al. 2020; Chaffee et al. 2021; Wittenberg et al. 2020; Herman and Tarran 2020).

Nicotine is the main toxic pollutant in the waste from tobacco cultivation, and improper handling can leave high levels of it in the environment (Cheng et al. 2021). Nicotine is considered as a toxic discharge; for example, European Union Regulations classify tobacco waste as “toxic and harmful” when the nicotine content exceeds 0.05% (*w*/*w*) (Novotny and Zhao 1999). Therefore, the degradation of nicotine in tobacco and its waste is an essential environmental concern to reduce the risk of ecosystem toxicity.

Tobacco is an important economic crop in many countries (Zou et al. 2021). Of the six trillion cigarettes consumed globally each year, four and a half trillion are disposed of somewhere in the environment (Araújo and Costa 2019). Tons of tobacco waste from plant to product need to be correctly treated (Novotny and Zhao 1999; Lam et al. 2022). At present, the main methods of treating tobacco waste are (1) direct incineration, causing air pollution; (2) extraction and utilization of useful substances, causing problems concerning the disposal of residual waste; and (3) resource utilization and harmless treatment technologies, such as composting or reconstituted tobacco leaf technology (Di et al. 2022; Cai et al. 2016). Some physical and chemical methods are used for tobacco waste treatment, but these have negative effects on the subsequent utilization of tobacco resources. In contrast, the biological treatments, such as nicotine degradation mediated by microbes or enzymes, are eco-friendly and low-cost methods (Zhang et al. 2022). Many nicotine-degrading microorganisms have been used for nicotine degradation in tobacco waste. At present, most of them are bacteria, including *Pseudomonas*, *Arthrobacter*, *Paleobacter*, and *Agrobacterium* (Liu et al. 2015; Zhong et al. 2010; Zhang et al. 2022; Guo et al. 2019). Nicotine-degrading bacteria (NDB) are mostly used in the composting of tobacco waste (Ye et al. 2017; Wang et al. 2013), the treatment of tobacco factory wastewater (Sabzali et al. 2012), and as a bacterial agent to remediate nicotine-contaminated soil (Shang et al. 2017). For example, a nicotine-degrading bacterium, *Arthrobacter histidinolovorans* EA-17, was added to the compost where tobacco straw as raw material was decomposed into organic fertilizer and the nicotine degradation rate was increased by 74.5% in comparison with the control (Wang et al. 2015).

Previous studies have demonstrated three main pathways of bacterial nicotine degradation. In the pyridine degradation pathway represented by the Gram-positive bacteria *Arthrobacter*, the main degradation enzymes include nicotine dehydrogenase, 6-hydroxynicotine oxidase, 6-hydroxypseudooxynicotine dehydrogenase, 2,6-dihydroxypseudooxynicotine hydrolase, 2,6-dihydroxypyridine 3-monooxygenase, and nicotine blue oxidoreductase (Sachelaru et al. 2005; Mihasan et al. 2007; Schenk et al. 1998; Deay et al. 2020). In the pyrrolidine pathway represented by the Gram-negative bacteria *Pseudomonas*, the most important enzymes are nicotine oxidoreductase and 3-succinoylpyridine monooxygenase (Hu et al. 2019; Xia et al. 2018). A variant of the pyridine and pyrrolidine pathways, named the VPP pathway (Huang et al. 2020), is found in the bacteria *Agrobacterium* sp. S33 (Shang et al. 2021), *Stenotrophomonas geniculata* N1 (Liu et al. 2014), and so on. Nonetheless, the degradation mechanisms of many NDB have not been elucidated so far, such as in *Rhizobium* spp. and *Bacillus* spp. (Mu et al. 2020; Liu et al. 2015; Zhang et al. 2022). Isolation and application of high-efficiency NDB are still important for nicotine degradation in environmental and human health; for example, the colonization of nicotine-degrading bacterium *Bacteroides xylanisolvens* in the gut reduced tobacco smoking-exacerbated non-alcoholic fatty liver disease (Chen et al. 2022).

This paper aims to investigate NDB diversity and the relationship between the abundance of nicotine-degrading genes and nicotine concentrations in tobacco cultivation soils. Various NDB were isolated from tobacco rhizosphere soil and two efficient NDB were successfully applied in the composting of tobacco waste. In addition, nicotine-degrading genes and their function in soil were analyzed by using metagenomic methods.

## Materials and methods

### Soil sampling

The tobacco rhizosphere soils were collected from Enshi, Hubei Province, China (30° 18′ N 109° 23′ E). The soils were sampled between the flourishing and mature periods of tobacco growth. Five treatments with six replicates in completely randomized blocks were established. The treatments were as follows: (1) Control, non-tobacco planting soil; (2) Site I, planting tobacco in the first year; (3) Site II, successive cropping with unhealthy tobacco; (4) Site III, successive cropping with healthy tobacco; and (5) Site IV, successive cropping with lethal tobacco. A total of 30 soil samples were collected and sieved with 2 mm mesh. Each soil sample was divided into four parts. Three of them were, respectively, used for the detection of nicotine concentrations, soil physicochemical properties, and metagenomic sequencing by Guangzhou MAGI Gene Sequencing Co. The last one was stored at – 80 ℃ for other experiments. In addition, NDB were isolated from one mixed tobacco planting soil sample.

### Determination of nicotine concentrations in samples

Nicotine concentrations in liquid media were measured with a UV spectrophotometer (Wang et al. 2015). The cultural samples were diluted with 0.05 mol/L HCl solution, and then the absorbance value at 259 nm wavelength was detected. Nicotine concentrations in soil and organic fertilizer were measured with high-performance liquid chromatography (HPLC) (Jablonski et al. 2006). First, nicotine was extracted using the following procedure. One gram (DW) of tobacco organic fertilizer sample was added to 2 mL 1 mol/L sodium hydroxide solution, and the pH of the sample was adjusted to be alkaline. Then, 3 mL ethanol was added to the solution, which was mixed and kept under 50 ℃ for 30 min and followed by ultrasonic treatment for 30 min. After that, the extracted nicotine was filtered with a 0.22 μm filter membrane. Finally, nicotine concentration in each sample was determined with HPLC (Shimadzu LC-20AT, Japan Shimadzu Company).

### Isolation and identification of nicotine-degrading bacteria

Nicotine selection medium (with nicotine as the sole carbon source) (Zhao et al. 2012; Ruan et al. 2005) was as follows: (NH_4_)_2_SO_4_ 2.0 g/L, MgSO_4_·7H_2_O 0.2 g/L, CaCl_2_·2H_2_O 0.01 g/L, FeSO_4_·7H_2_O 0.001 g/L, Na_2_HPO_4_·12H_2_O 2.33 g/L, NaH_2_PO_4_·2H_2_O 0.55 g/L. Nicotine was purchased from Desit Biological Company, China.

The soil sample was serially diluted with sterile water and then dilutions were spread on a nicotine selection medium and incubated at 28 °C aerobically. After 2 days, single colonies were picked and further purified. The 16S rRNA gene was amplified with the primer pair 27F/1492R. The 16S rRNA gene sequences of all NDB pair-end sequencing were carried out in an Illumina Hiseq PE150 system by Tsingke Biotechnology Co., Ltd. After the sequencing results were assembled, the 16S rRNA genes were analyzed on the EZbiocloud website (www.ezbiocloud.net). The sequences were deposited in the National Microbiology Data Center (https://nmdc.cn/). The accession numbers are NMDCN000175N ND1-NMDCN0001769 ND43.

### Capacity of nicotine degradation in medium

Each strain involved in nicotine degradation was individually inoculated into 5 mL LB medium and incubated at 28 ℃ with 150 rpm shaking. When OD_600_ reached 0.8–1.0, cells were inoculated into a 50 mL medium with the presence of 1.0 g/L nicotine. Culture samples were collected every 12 h for nicotine concentration measurement. Last, the nicotine concentrations of the samples and the nicotine-degrading rate of each strain were calculated, and the high-efficiency NDB were screened for the next experiments.

### Effects of temperature and nicotine concentration on nicotine degradation

Strains ND6 and ND16 were selected as the high-efficiency NDB based on the results described in “Capacity of nicotine degradation in medium” Sect. In order to more clearly compare the nicotine-degrading ability and the phenotypic differences during nicotine degradation, a classical strain *Pseudomonas putida* S16 with a known degradation pathway from Dr. Ping Xu in Shanghai Jiao Tong University (Yu et al. 2011) was used as control.

Assessments of the nicotine-degrading characteristics of these three strains were performed in nicotine medium under 28 ℃ and 37 ℃ as well as nicotine concentration gradient as 0.5 g/L, 1.0 g/L, and 2.0 g/L. Three parallel experiments were carried out in each experimental group. Samples were taken regularly every 12 h during the cultivation process to determine bacterial growth and nicotine-degrading capacity.

### Genome sequencing and nicotine-degrading pathway analysis of strains ND6 and ND16

Genome pair-end sequencing was carried out in an Illumina Hiseq system by Wuhan Carboncode Bioinformatics Co. Ltd., China. Genome assembly was conducted using SPAdes v3.11.1 (http://cab.spbu.ru/software/spades/) software with filtered reads. The genes of the two strains were annotated using the eggNOG and GO databases. The 16S rRNA gene and whole-genome sequences of strains ND6 and ND16 were aligned against all the related sequences from the Type (Strain) Genome Server (https://tygs.dsmz.de). Phylogenetic trees were constructed using the genome BLAST distance phylogeny method. The genomic average nucleotide identity and DNA–DNA hybridization values were obtained from EzBioCloud (https://www.ezbiocloud.net) and the Type (Strain) Genome Server.

KEGG analysis was performed on the predicted genes of ND6 and ND16, and the metabolic pathways of nicotine degradation in these strains were annotated in comparison with the known nicotine metabolic pathways (https://www.kegg.jp/pathway/map=map00760&keyword=nicotine).

### Application of efficient NDB in fermented compost of tobacco waste

First, LB was used to cultivate strains ND6, ND16, and S16, and then the bacterial solution was inoculated into the compost fertilizer from tobacco waste from the Enshi Tobacco Company, Hubei Province. The total inoculum amount of bacterial solution in each experimental group was 10%, about 10^7^ cfu/g. The same volume of bacteria-free medium was added to the control. Each experiment was performed on three replicates. The control samples were tested in the absence of additional bacterial cells, then all groups were placed in a 28 ℃ incubator for 12 days, and samples were taken once every 6 days. The nicotine concentrations of dry weight were determined as described in Determination of nicotine concentrations in samples.

### Correlation analysis between nicotine concentrations and abundance of nicotine-degrading genes in soil

The metagenomic data determination was performed by Guangzhou MAGI Gene Sequencing Co. The abundance of nicotine-degrading genes was calculated with the metagenomic data. Redundancy analysis (RDA), a multivariate direct gradient analysis method, was calculated using Canoco version 5.0 to elucidate the relationships between soil physicochemical properties, enzyme activities, and the relative abundance of nicotine degradation-related genes.

### Statistical analysis

All data required for between-group significance were statistically analyzed. The software SPSS Statistics 26.0 was used for data comparison. Different letters were used to represent differences between groups.

## Results

### Multiple nicotine-degrading bacteria in tobacco rhizosphere soil

**Fig. 1:**
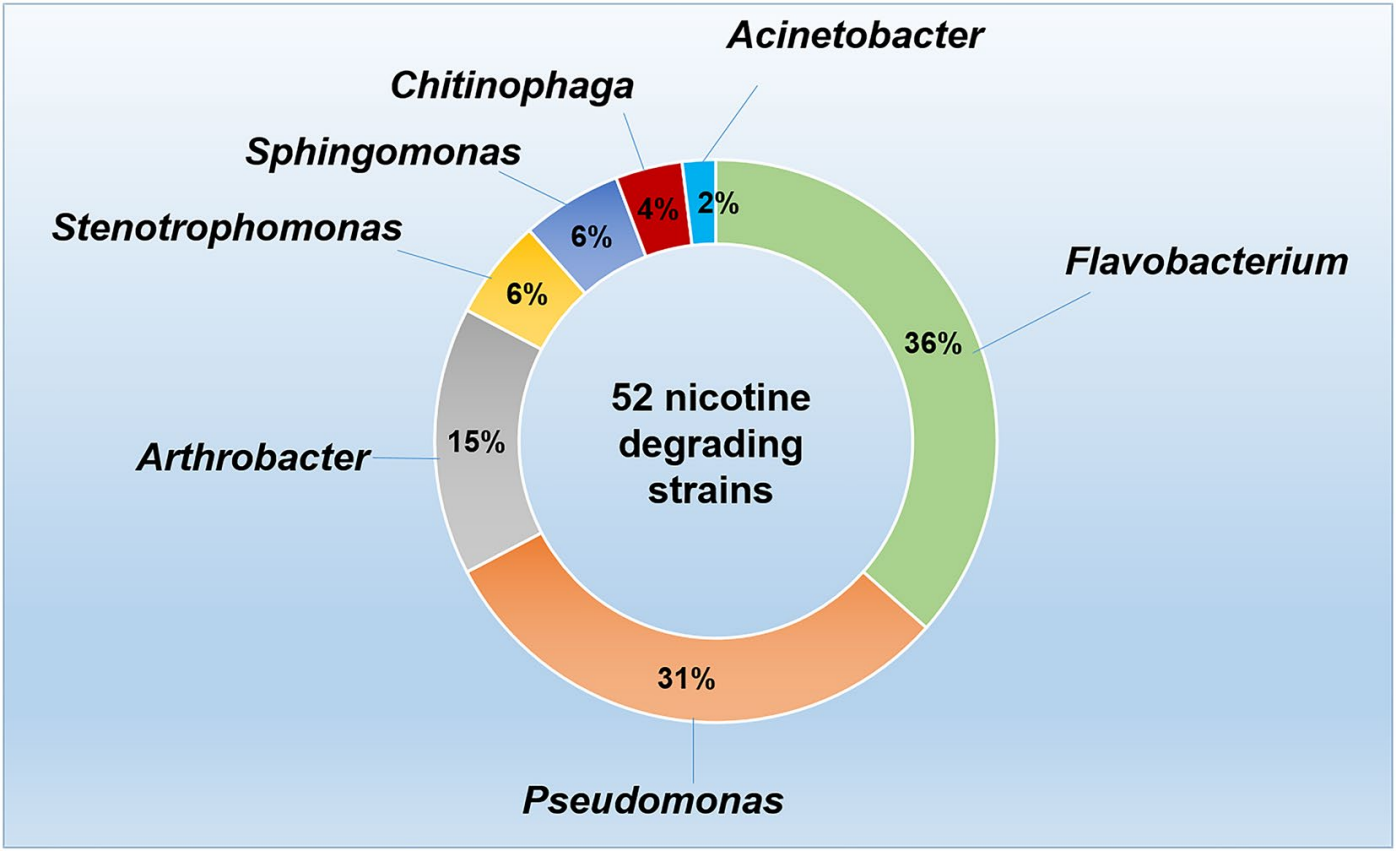
Diversity of nicotine-degrading bacteria isolated from the tobacco rhizosphere soil

From the tobacco cultivation rhizosphere soil, 52 NDB strains were obtained after separation and purification under a plate with 1.0 g/L of nicotine serving as the sole carbon source. Based on the results of the 16S rRNA gene sequence alignment, these 52 strains were found to belong to seven genera, including *Flavobacterium*, *Pseudomonas*, *Arthrobacter*, *Stenotrophomonas*, *Sphingomonas*, *Chitinophaga,* and *Acinetobacter* (Fig. 1A). The most dominant genera were *Flavobacterium*, *Pseudomonas,* and *Arthrobacter*, accounting for 36.54%, 30.77%, and 15.38%, respectively. It is interesting that the strains from genera *Flavobacterium* and *Chitinophaga* had not been previously reported as NDB.

All these strains were then cultured in a liquid medium with an additional 1.0 g/L of nicotine. During 7-day incubation, the nicotine-degrading rates of these strains shifted from 20 to 98%, in which strains ND6 and ND16 showed the highest nicotine-degrading rates of 97% and 98%, respectively. Consequently, strains ND6 and ND16 were selected for subsequent experiments including genome sequencing, species identification, nicotine-degrading ability and pathway analysis, and application in compost fermentation of tobacco waste.

### Taxonomic identification of efficient NDB ND6 and ND16

**Fig. 2:**
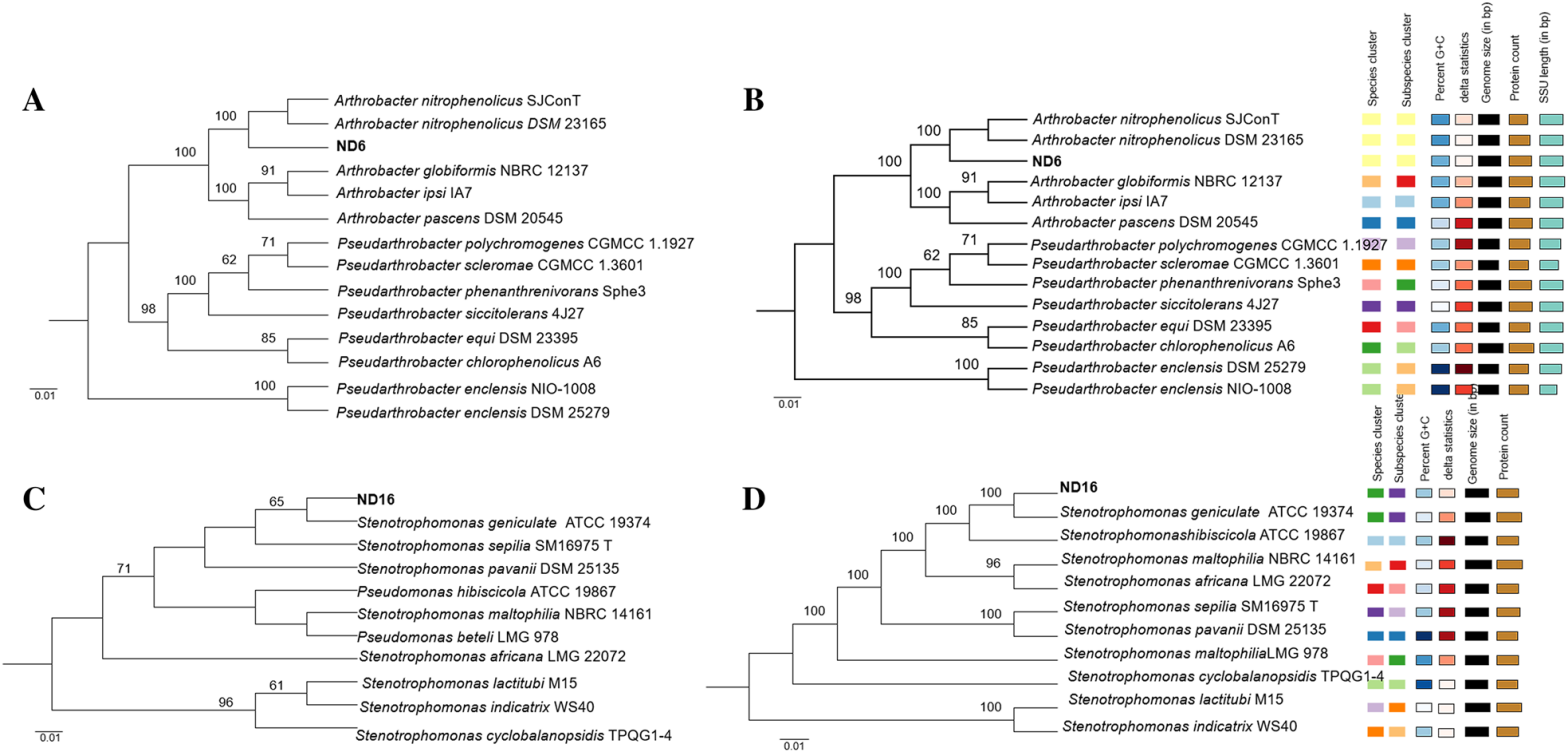
Phylogenetic trees of strains ND6 (**A**, **B**) and ND16 (**C**, **D**) based on 16S rRNA gene sequences (**A**, **C**) and genome (**B**, **D**)

The 16S rRNA gene sequence analysis of strain ND6 revealed that it belonged to the genus *Arthrobacter*, with the highest similarity (99.2%) to the species *Arthrobacter nitrophenolicus* SJConT. As shown in Fig. 2A and B, the phylogenetic trees based on 16S rRNA gene sequences and genome showed strain ND6 is close to *A. nitrophenolicus*. Moreover, the average nucleotide identity (ANI) value and DNA–DNA hybridization (DDH) value between ND6 and *A. nitrophenolicus* SJConT were 95.9% and 86.2%, respectively (Additional file 1: Table S1), meeting the cut-off value for the same species (Chun et al. 2018). Consequently, strain ND6 was identified as *Arthrobacter nitrophenolicus*. Likewise, strain ND16 was identified as *Stenotrophomonas geniculata* (Fig. 2C and D, Additional file 1: Table S2).

### Nicotine metabolic pathway analysis of strains ND6 and ND16

**Fig. 3:**
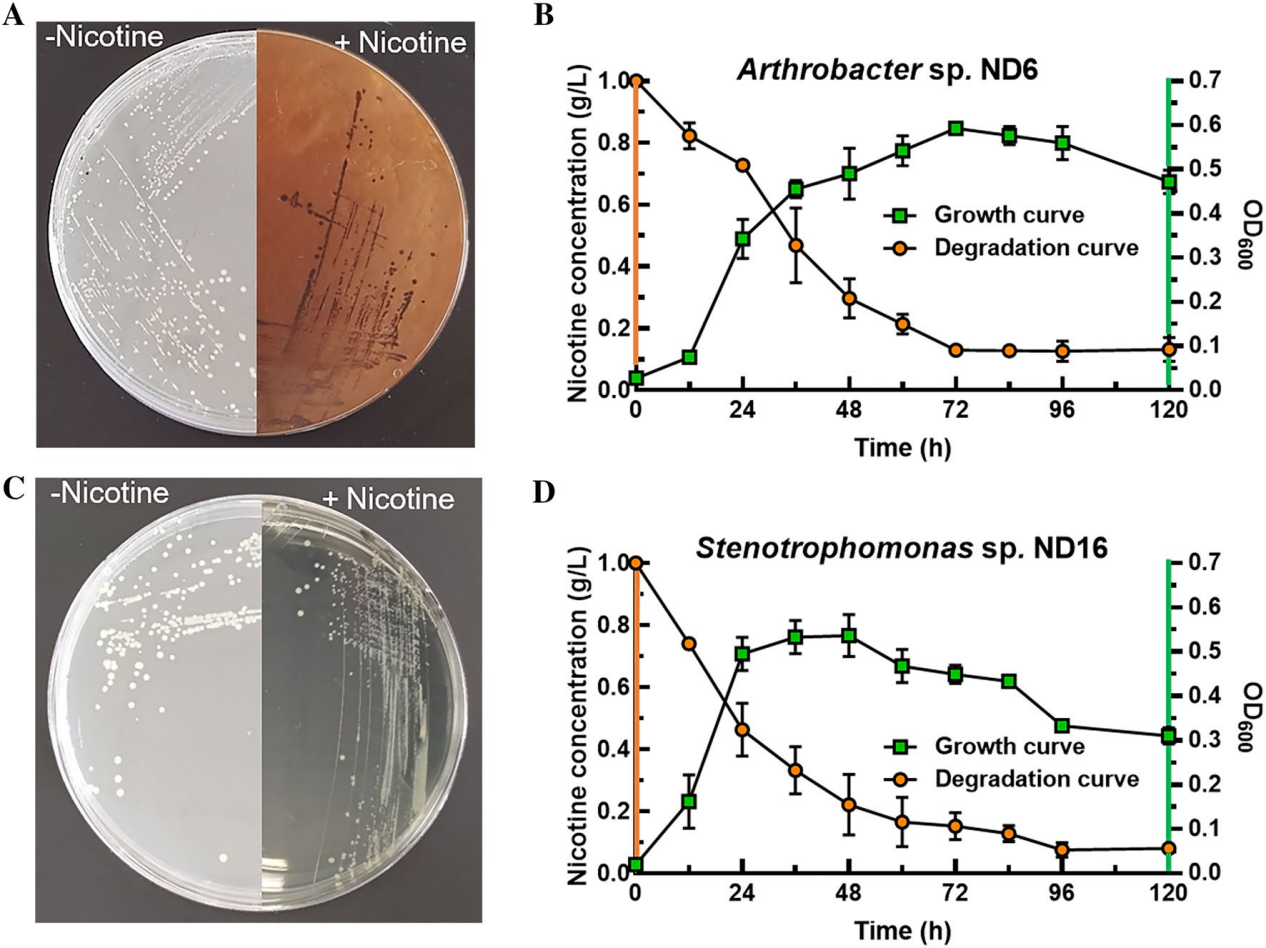
Strains ND6 and ND16 growing on plate and liquid medium with 1.0 g/L of nicotine as sole carbon source. **A**, **C** The strains grew on the plate; **B**, **D** growth curves and nicotine-degrading curves under 28 ℃

*A. nitrophenolicus* ND6 and *S. geniculata* ND16 all grew well in nicotine medium with an additional 1.0 g/L of nicotine (Fig. 3). Strain ND6 showed deep blue color at an early stage and then gradually became brown (Fig. 3A and Additional file 1: Fig. S3), degrading 87% of nicotine after 3 days (Fig. 3B). In contrast, strain ND16 did not change color over time, degrading 92.6% of nicotine after 5 days (Figs. 3C and D).

The nicotine metabolic pathways of the two strains are shown in Additional file 1: Figs. S1 and S2. It was found that five key genes of the pyridine pathway are annotated in strain ND6, including nicotine dehydrogenase, (S)-6-hydroxynicotine oxidase, 6-hydroxypseudooxynicotine dehydrogenase, 2,6-dihydroxypseudooxynicotine hydrolase, and nicotine blue oxidoreductase (Additional file 1: Fig. S1). The color change from blue to brown was consistent with the color phenotype of the pyridine pathway (Fig. 3A and Additional file 1: Fig. S3). Therefore, strain ND6 degrades toxic nicotine into nutrients for its own growth through the pyridine pathway. In contrast, strain ND16 was not annotated any key gene involved in the nicotine metabolic pathway, nor did it have any color change in nicotine-containing incubation over time (Figs. 3C, Additional file 1: Figs. S2 and S3). The color change was different from reported pathways, including pyridine pathway’s change from blue to brown, the green color of the pyrrolidine pathway, and the deep brown color of the VPP pathway. Consequently, both genotype and phenotype indicated that strain ND16 may have a novel nicotine-degrading pathway.

### Effects of temperature on nicotine degradation in strains ND6 and ND16

A known efficient nicotine-degrading bacterium *P. putida* S16 was used as the control for comparison of nicotine-degrading ability with an additional 1.0 g/L of nicotine under 28 ℃ or 37 ℃.

**Fig. 4:**
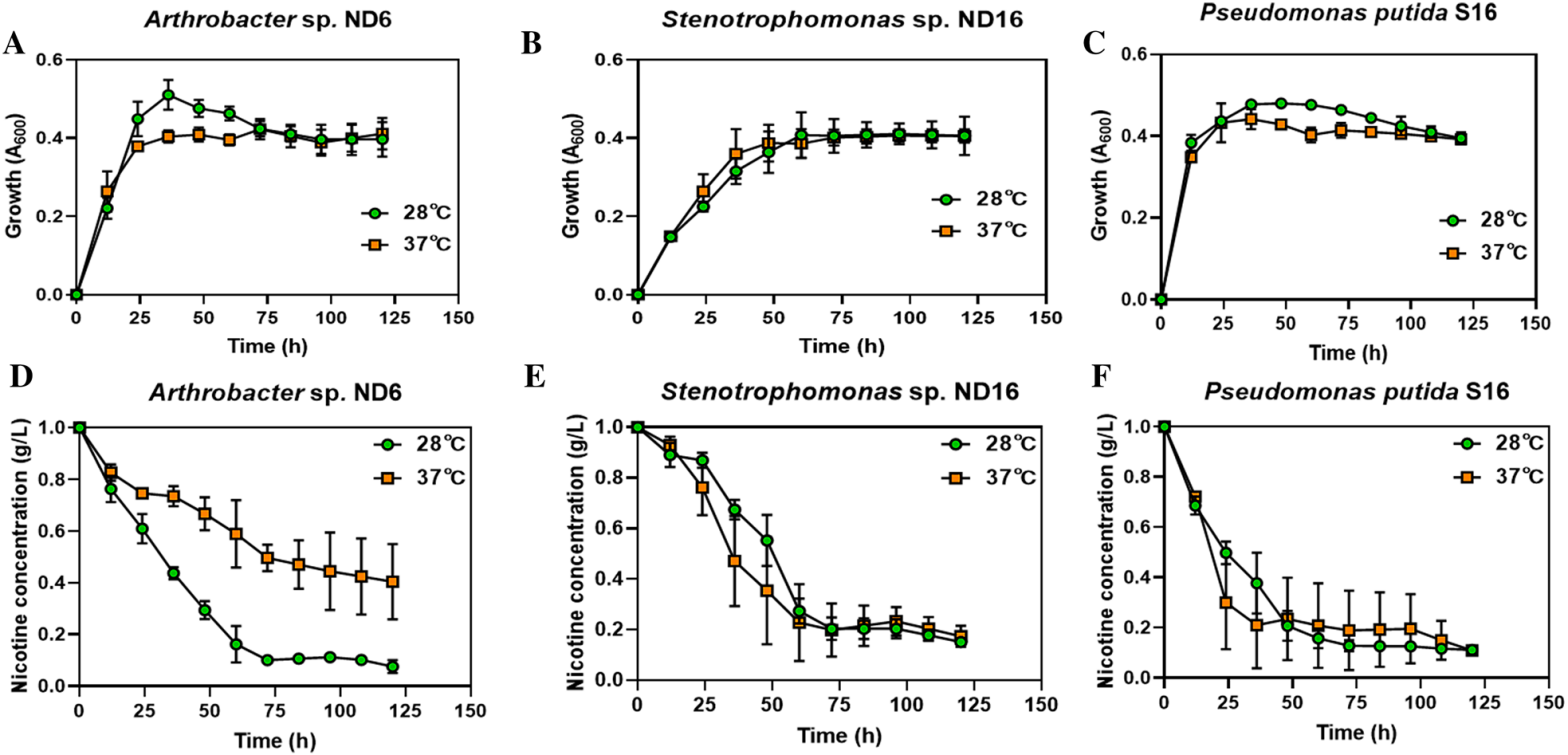
Effects of temperature on bacterial growth and nicotine degradation. Nicotine-degrading bacteria *Arthrobacter nitrophenolicus* ND6 (**A**, **D**), *Stenotrophomonas geniculata* ND16 (B, E), and control strain *Pseudomonas putida* S16 (C, F). **A**–**C** Growth curves of strains under different temperatures; **D**–**F** nicotine-degrading curves of strains under different temperatures

Strain *A. nitrophenolicus* ND6 grew rapidly during 0–24 h, and bacterial mass reached its maximum value at 36 h under 28 ℃. In contrast, the biomass was lower under 37 ℃ than under 28 ℃, and the nicotine-degrading ability declined significantly (Fig. 4A and D). The growth and degradation of strain *S. geniculata* ND16 did not show a significant difference under 28 ℃ and 37 ℃ (Fig. 4B and E). The control strain of *P. putida* S16 showed a weak decrease in growth and degradation under 37 ℃ in comparison with 28 ℃ incubation (Fig. 4 C and F).

When the temperature was 28 ℃, strain ND6 had the best growth, followed by strains S16 and ND16. At 37 ℃, strain S16 had the best growth, followed by strains ND6 and ND16. The final biomass values shown by the OD_600_ values of the three strains were very close at 0.4 under the two temperatures. Temperature of 28 ℃ and 37 ℃ only affected the growth rate of these three strains at the early and middle stages, but not the final biomass.

Besides growth, all these three strains were able to degrade more than 80% of 1.0 g/L nicotine under 28 ℃; ND6 had the strongest degradation capacity of more than 90% (Fig. 4D–F). When the temperature was 37 ℃, the nicotine degradation ability of ND6 significantly decreased to 60%, while the degradation ability of ND16 and S16 was almost unaffected at different temperatures. Therefore, temperature had the greatest effect on the degradation ability of ND6, but almost no effect on ND16.

### Effects of nicotine concentrations on degradation in strains ND6 and ND16

**Fig. 5:**
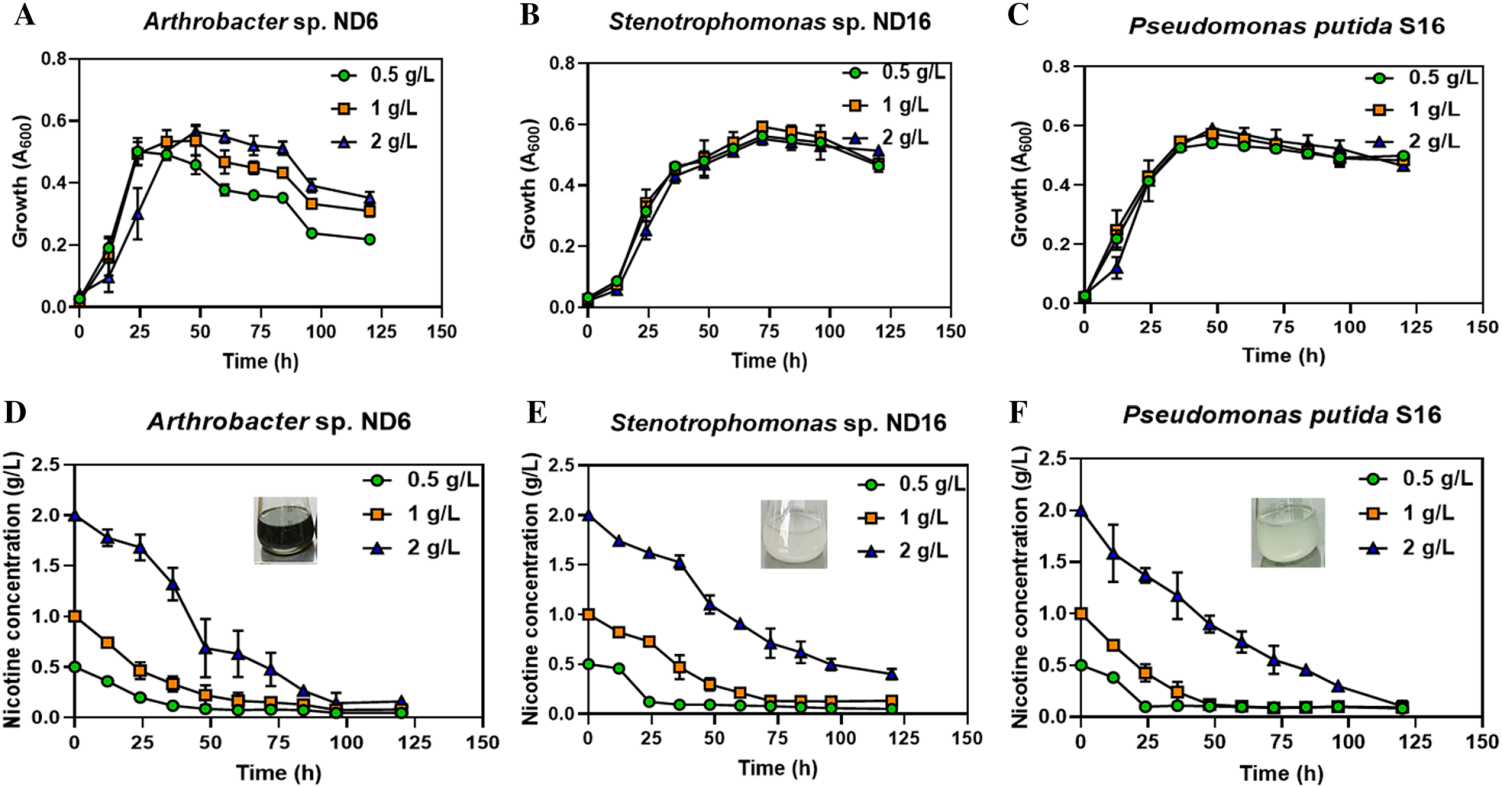
Effects of nicotine concentrations on bacterial growth and nicotine degradation. **A**–**C** Growth curves of tree strains under different nicotine concentrations; **D**–**F** nicotine-degrading curves and colors of three strains under different nicotine concentrations

Strains *A. nitrophenolicus* ND6, *S. geniculate* ND16, and *P. putida* S16 all grew well in a nicotine medium with a nicotine concentrations of 0.5–2.0 g/L at 28 ℃ (Fig. 5). The nicotine concentrations had the greatest effect on the growth of strain ND6, which showed a significant delay in growth under 2.0 g/L of nicotine within 36 h, however, the growth rate was accelerated after 36 h with the higher biomass than that under nicotine concentrations 0.5 g/L or 1.0 g/L. In contrast, the growth of ND16 and S16 only showed a weak delay at the exponential phase under 2.0 g/L of nicotine in comparison with growth under 0.5 g/L and 1.0 g/L.

Under 0.5 g/L of nicotine concentration, strains ND16 and S16 rapidly degraded 80% of nicotine within 24 h, which was faster than ND6 (Fig. 5). Under 1.0 g/L of nicotine concentration, the fastest nicotine-degrading rate of 88% was observed in strain S16 within 48 h, followed by 78% in strain ND6 and 70% in ND16. When nicotine concentration reached 2.0 g/L, strain ND6 had the highest degrading rate of 95% within 96 h; however, the degrading rates of strain S16 and ND16 were 73% and 56%, respectively. Strain ND16 degraded nicotine faster than ND6 under 0.5 g/L of nicotine; in contrast, ND6 had the higher nicotine-degrading rate than ND16 under more than 1.0 g/L. The growth and nicotine-degrading capacity of the two strains were close to the reported control strain S16. Therefore, strains ND6 and ND16 were utilized in the subsequent nicotine degradation in compost fertilizer fermented from tobacco waste.

### Application of NDB in nicotine degradation of tobacco compost

**Fig. 6:**
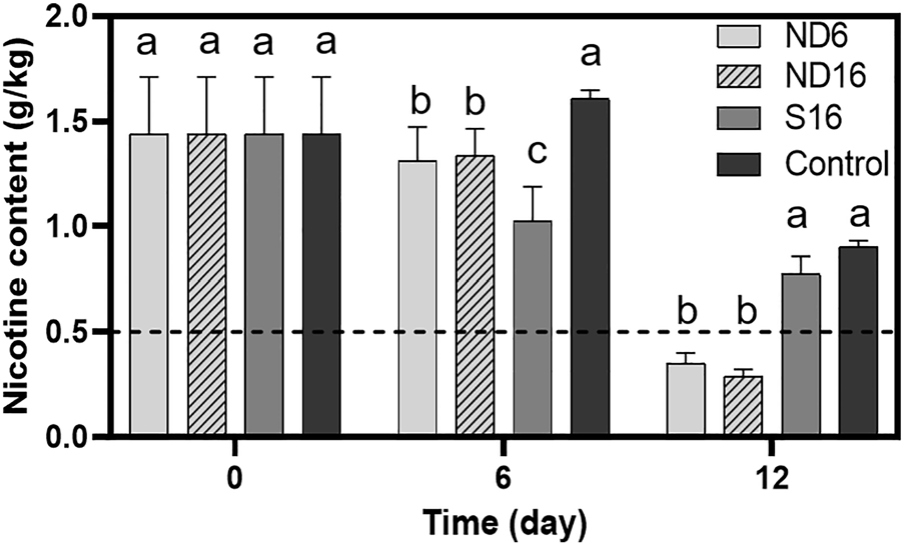
Addition of nicotine-degrading bacteria ND6 and ND16 on nicotine degradation in compost fertilizer fermented from tobacco waste

Strains *A. nitrophenolicus* ND6, *S. geniculate* ND16, and *P. putida* S16 were used in nicotine degradation of compost fertilizer fermented from tobacco waste. Un-inoculated compost served as a control with native NDB. The initial nicotine concentration in the tobacco compost was approximately 1.5 mg/g (DW). After 12 days of cultivation, the concentration of nicotine in each group of compost significantly decreased (Fig. 6). The nicotine concentration in compost significantly decreased from 1.5 to 0.35 mg/g, 0.29 mg/g, and 0.77 mg/g with additional strains ND6, ND16, and S16, respectively, which was lower than that in the control by 0.9 mg/g. In particular, the nicotine concentrations in compost fertilizer inoculated with strains ND6 and ND16 were below the European Union Regulations threshold of 0.5 mg/g (Novotny and Zhao 1999).

### Correlation between soil nicotine concentrations and abundance of nicotine degradation genes

**Fig. 7:**
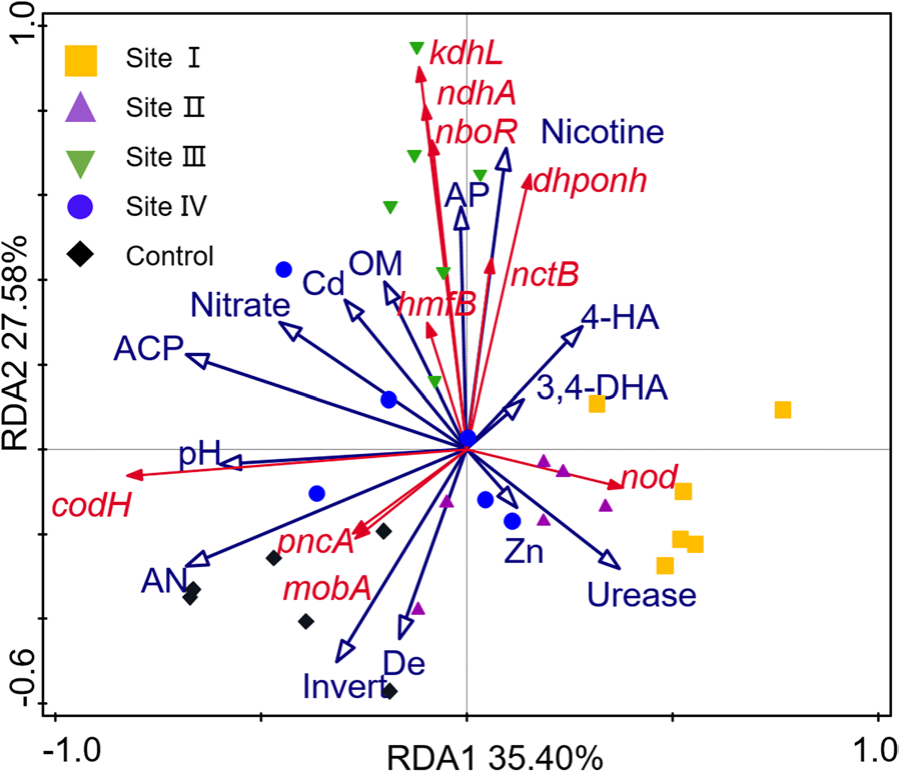
Redundancy analysis of the relationships among nicotine concentrations, abundance of nicotine-degrading genes and some soil properties in tobacco rhizosphere soils. *codH* aerobic carbon-monoxide dehydrogenase gene, *ndhA* nicotine dehydrogenase subunit gene, *kdhL* 6-hydroxypseudooxynicotine dehydrogenase subunit gene, *hmfB* 2-furoyl-CoA dehydrogenase gene, *mobA* molybdenum cofactor cytidylyltransferase gene, *nboR* nicotine blue oxidoreductase gene, *dhponh* 2,6-dihydroxypseudooxynicotine hydrolase gene, *nctB* (S)-6-hydroxynicotine oxidase gene, *pncA* nicotinamidase gene, *nod* nicotine oxidoreductase gene, *De* dehydrogenase, *ACP* acid phosphatase, *AP* organic phosphorus, *AN* alkali hydrolyzed nitrogen, *OM* organic matter, *4-HA* 4-Hydroxybenzoic acid, *3,4-DHA* 3,4-dihydroxyphenylacetic acid, *Cd* cadmium, *Zn* zinc

Nicotine concentrations was varied from 0.0 to 77.7 mg/kg in tobacco rhizosphere soil in order of Control, Site I, Site II, Site IV, and Site III (Fig. 7). Ten nicotine-degrading genes were annotated from the metagenome. They are the genes of nicotine dehydrogenase subunit (NdhA), 6-hydroxypseudooxynicotine dehydrogenase subunit (KdhL), nicotine blue oxidoreductase (NboR), 2-furoyl-CoA dehydrogenase (HmfB), 2,6-dihydroxypseudooxynicotine hydrolase (Dhponh), (S)-6-hydroxynicotine oxidase (NctB), nicotine oxidoreductase (Nod), aerobic carbon-monoxide dehydrogenase (CodH), nicotinamidase (PncA), and molybdenum cofactor cytidylyltransferase (MobA). Among the ten nicotine-degrading genes, *ndhA*, *nctB*, *kdhL*, *nboR*, and *dhponh* are the key nicotine-degrading genes in the pyridine pathway, and *ndhA* and *nctB* are also the key genes of the VPP pathway. The rest of the genes have multiple functions besides of nicotine degradation.

RDA clearly showed that nicotine concentrations were positively and strongly correlated with the abundance of the genes *ndhA*, *nctB*, *kdhL*, *nboR*, *hmfB,* and *dhponh* (Fig. 7). However, the abundance of *nod* and *codH* had no correlation with nicotine concentrations. Moreover, the abundance of *pncA* and *mobA* had a negative correlation with nicotine concentrations.

## Discussion

The reported NDB in recent years mainly include species from *Arthrobacter*, *Pseudomonas*, *Acinetobacter*, *Ochrobactrum*, and *Agrobacterium*, among which fewer genomes have been sequenced (Zhang et al. 2022). Our research confirmed at least seven genera of NDB in the rhizosphere soil of tobacco plantings. To our knowledge, the genera *Flavobacterium* and *Chitinophaga* have not been previously reported as NDB. It is interesting that *Flavobacterium* species were the most abundant nicotine-degrading strains in the tobacco rhizosphere soil. Many species involved in nicotine degradation have not received sufficient attention, possibly due to their low nicotine-degrading capacity, such as in this case. Therefore, it is possible that NDB with high nicotine-degrading ability will be found in the future.

Investigation of nicotine-degrading pathways is an important field, although some pathways, including the pyridine pathway, pyrrolidine pathway, and their combined VPP pathway, have been elucidated in *Arthrobacte*r, *Pseudomonas,* and *Agrobacterium* (Hu et al. 2019; Liu et al. 2015; Shang et al. 2021). In this study, both genomic annotation and phenotype of color change indicated that *S. geniculata* ND16 may have a novel nicotine-degrading pathway (Additional file 1: Figs. S2 and S3). In particular, the known nicotine degradation enzymes 6-hydroxypseudooxynicotine amine oxidase and 6-hydroxy-3-succinoylpyridine hydroxylase in same species *S. geniculata* N1 were not annotated in ND16 (Wang et al. 2019). The nicotine metabolic pathways have not been elucidated in other bacteria, for example, for the *Flavobacterium* and *Chitinophaga* species in this study. As a result, this situation limits deep investigation of nicotine-degrading genes and analysis of their function in the environment. Nonetheless, we have explored the abundance of these effective nicotine-degrading genes in soil and tried to describe the relationship between gene abundance, nicotine-degrading status, and nicotine concentrations. Interestingly, the soil nicotine concentrations correlated well with the abundance of five nicotine-degrading genes that belonged to the pyridine and VPP pathways, encompassing *ndhA*, *nctB*, *kdhL*, *dhponh,* and *nboR* (Fig. 7). In addition, these five abundant genes represent the initial, middle, and concluding reactions of nicotine metabolism (Li et al. 2016; Mihasan et al. 2007; Sachelaru et al. 2005), implying that nicotine can be completely degraded into non-toxic nutrients in a timely manner in the tobacco rhizosphere soil. Therefore, there exists the potential to utilize these key genes to monitor nicotine metabolism mediated by bacteria in the environment and to inoculate nicotine-contaminated environments with them to eliminate nicotine toxicity. However, some nicotine degradation-related genes, such as *nod* and *codH,* were not correlated with nicotine concentrations, possibly because of their multiple functions. For example, Nod is both a nicotine oxidoreductase and an amine oxidase. However, further study is needed because none of the genes of the pyrrolidine pathway have been detected in the soil, which may have three possibilities. One is lower gene abundance of pyrrolidine pathway, such as in *Pseudomonas*. The second reason is that most nicotine-degrading genes have not been elucidated as mentioned above. Lastly, it may be related to sequencing depth.

In this study, we also successfully applied NDB to degrade nicotine in compost fertilizer fermented from tobacco waste (Fig. 6), showing the nicotine concentration was below the European Union Regulations threshold of 0.5 mg/g. This provides an environmentally friendly method of producing safe organic fertilizer by using huge amounts of tobacco waste, such as straw, waste tobacco leaves, and abandoned tobacco shreds. Notably, it is necessary to isolate or genetic-modify more efficient and tolerant nicotine-degrading microbes in compost fermentation to rapidly decrease the nicotine concentration since high temperature or limited oxygen depresses microbial growth and nicotine degradation during the composting process.

## Conclusion

In this study, multiple and diverse NDB have been isolated from tobacco rhizosphere soil and two high-efficiency NDB *A. nitrophenolicus* ND6 and *S. geniculata* ND16 were successfully applied to degrade nicotine in the compost fertilizer of tobacco waste. In particular, strain ND16 may follow a new pathway of nicotine degradation according to genomic analysis and color change. It is noteworthy that to isolate more NDB and elucidate their nicotine-degrading pathways and to apply these NDB to eliminate nicotine toxicity in nicotine-contaminated environments. In addition, five key genes, *ndhA*, *nctB*, *kdhL*, *nboR*, and *dhponh*, represent the whole process of nicotine degradation, and their abundance positively correlated with nicotine concentrations in tobacco planting soil (*p* < 0.05). This would offer a more promising way to utilize these key genes to monitor nicotine metabolism mediated by bacteria in the environment.

### Supplementary Information

**Additional file 1: Fig. S1.** Nicotine metabolic pathway of bacterium *Arthrobacter* *nitrophenolicus *ND6 showing in the red box at the lower left corner. **Fig. S2.** Nicotine metabolic pathway of bacterium *Stenotrophomonas* *geniculata* ND16 showing in the red box at the lower left corner. **Fig. S3.** Color change showing by NDB under different temperatures (A) and nicotine concentrations (B). **Table S1.** The genomic average nucleotide identity (ANI) and digital DNA–DNA hybridization (dDDH) values between *Arthrobacter *sp. ND6 with affiliated species. **Table S2.** The genomic average nucleotide identity (ANI) and digital DNA–DNA hybridization (dDDH) values between *Stenotrophomonas *sp. ND16 with affiliated species.

## Data Availability

Not applicable.

## References

[CR1] Araújo MCB, Costa MF (2019). From plant to waste: the long and diverse impact chain caused by tobacco smoking. Int J Environ Res Public Health.

[CR2] Cai JX, Li B, Chen CY, Wang J, Zhao M, Zhang K (2016). Hydrothermal carbonization of tobacco stalk for fuel application. Bioresour Technol.

[CR3] Chaffee BW, Couch ET, Vora MV, Holliday RS (2021). Oral and periodontal implications of tobacco and nicotine products. Periodontol 2000.

[CR4] Chen B, Sun LL, Zeng GY, Shen Z, Wang K, Yin LM, Xu F, Wang PC, Ding Y, Nie QX, Wu Q, Zhang ZW, Xia JL, Lin J, Luo YH, Cai J, Krausz KW, Zheng RM, Xue YX, Zheng MH, Li Y, Yu CH, Gonzalez FJ, Jiang CT (2022). Gut bacteria alleviate smoking-related NASH by degrading gut nicotine. Nature.

[CR5] Cheng YD, Bai YX, Jia M, Chen Y, Wang D, Wu T, Wang G, Yang HW (2021). Potential risks of nicotine on the germination, growth, and nutritional properties of broad bean. Ecotoxicol Environ Saf.

[CR6] Chun J, Oren A, Ventosa A, Christensen H, Arahal DR, Costa MS, Rooney AP, Yi H, Xu XW, Meyer SD (2018). Proposed minimal standards for the use of genome data for the taxonomy of prokaryotes. Int J Syst Evol Microbiol.

[CR7] Deay DO, Colvert KK, Gao F, Seibold S, Goyal P, Aillon D, Petillo PA, Richter ML (2020). An active site mutation in 6-hydroxy-l-Nicotine oxidase from *Arthrobacter nicotinovorans* changes the substrate specificity in favor of (S)-nicotine. Arch Biochem Biophys.

[CR8] Di HH, Wang R, Ren XH, Deng JQ, Deng XH, Bu GJ (2022). Co-composting of fresh tobacco leaves and soil: an exploration on the utilization of fresh tobacco waste in farmland. Environ Sci Pollut Res Int.

[CR9] Guo XH, Xie CY, Wang LJ, Li QF, Wang Y (2019). Biodegradation of persistent environmental pollutants by *Arthrobacter* sp.. Environ Sci Pollut Res Int.

[CR10] Herman M, Tarran R (2020). E-cigarettes, nicotine, the lung and the brain: multi-level cascading pathophysiology. J Physiol.

[CR11] Hu HY, Wang LJ, Wang WW, Wu G, Tao F, Xu P, Deng ZX, Tang HZ (2019). Regulatory mechanism of nicotine degradation in *Pseudomonas putida*. Mbio.

[CR12] Huang HY, Shang JM, Wang SN (2020). Physiology of a hybrid pathway for nicotine catabolism in bacteria. Front Microbiol.

[CR13] Jablonski JE, Schlesser JE, Mariappagoudar P (2006). HPLC-UV method for nicotine, strychnine, and aconitine in dairy products. J Agric Food Chem.

[CR14] Lam J, Schneider J, Shadbegian R, Pega F, Claire SS, Novotny TE (2022). Modelling the global economic costs of tobacco product waste. Bull World Health Organ.

[CR15] Li HL, Xie KB, Yu WJ, Hu LJ, Huang HY, Xie HJ, Wang SN (2016). Nicotine dehydrogenase complexed with 6-Hydroxypseudooxynicotine oxidase involved in the hybrid nicotine-degrading pathway in *Agrobacterium tumefaciens* S33. Appl Environ Microbiol.

[CR16] Liu YH, Wang LJ, Huang KM, Wang WW, Nie XL, Jiang Y, Li PP, Liu SS, Xu P, Tang HZ (2014). Physiological and biochemical characterization of a novel nicotine-degrading bacterium *Pseudomonas geniculata* N1. PLoS ONE.

[CR17] Liu JL, Ma GH, Chen T, Hou Y, Yang SH, Zhang KQ, Yang JH (2015). Nicotine-degrading microorganisms and their potential applications. Appl Microbiol Biotechnol.

[CR18] McGrath-Morrow SA, Gorzkowski J, Groner JA, Rule AM, Wilson K, Tanski SE, Collaco JM, Klein JD (2020). The effects of nicotine on development. Pediatrics.

[CR19] Mihasan M, Chiribau CB, Friedrich T, Artenie V, Brandsch R (2007). An NAD(P)H-nicotine blue oxidoreductase is part of the nicotine regulon and may protect *Arthrobacter nicotinovorans* from oxidative stress during nicotine catabolism. Appl Environ Microbiol.

[CR20] Mu Y, Chen Q, Parales RE, Lu ZM, Hong Q, He J, Qiu JG, Jiang JD (2020). Bacterial catabolism of nicotine: catabolic strains, pathways and modules. Environ Res.

[CR21] Novotny TE, Zhao F (1999). Consumption and production waste: another externality of tobacco use. Tob Control.

[CR22] Ruan A, Min H, Peng X, Huang Z (2005). Isolation and characterization of *Pseudomonas* sp. strain HF-1, capable of degrading nicotine. Res Microbiol.

[CR23] Sabzali A, Nikaeen M, Bina B (2012). Performance evaluation of cigarette filter rods as a biofilm carrier in an anaerobic moving bed biofilm reactor. Environ Technol.

[CR24] Sachelaru P, Schiltz E, Igloi GL, Brandsch R (2005). An alpha/beta-fold C-C bond hydrolase is involved in a central step of nicotine catabolism by *Arthrobacter nicotinovorans*. J Bacteriol.

[CR25] Schenk S, Hoelz A, Krauss B, Decker K (1998). Gene structures and properties of enzymes of the plasmid-encoded nicotine catabolism of *Arthrobacter nicotinovorans*. J Mol Biol.

[CR26] Shang C, Chen A, Chen GQ, Li HK, Guan S, He JM (2017). Microbial biofertilizer decreases nicotine content by improving soil nitrogen supply. Appl Biochem Biotechnol.

[CR27] Shang JM, Wang X, Zhang M, Li LX, Wang RF, Huang HY, Wang SN (2021). An NAD-specific 6-Hydroxy-3-succinoyl-semialdehyde-pyridine dehydrogenase from nicotine-degrading *Agrobacterium tumefaciens* strain S33. Microbiol Spectr.

[CR28] Wang X, Tang L, Yao YL, Wang HX, Min H, Lu ZM (2013). Bioremediation of the tobacco waste-contaminated soil by *Pseudomonas* sp. HF-1: nicotine degradation and microbial community analysis. Appl Microbiol Biotechnol.

[CR29] Wang R, Shi HL, Chen SW (2015) Isolation of nicotine degradation bacterium strain and its application. China Academic Journal Electronic Publishing House. P 1212–1224

[CR30] Wang W, Zhu X, Liu X, Wu W, Xu P, Tang H (2019). Cloning and characterization the nicotine degradation enzymes 6-hydroxypseudooxynicotine amine oxidase and 6-hydroxy-3-succinoylpyridine hydroxylase in *Pseudomonas geniculata* N1. Int Biodeterior Biodegrad.

[CR31] Wittenberg RE, Wolfman SL, Biasi MD, Dani JA (2020). Nicotinic acetylcholine receptors and nicotine addiction: a brief introduction. Neuropharmacology.

[CR32] Xia ZY, Lei LP, Zhang HY, Wei HL (2018). Characterization of the ModABC molybdate transport system of *Pseudomonas putida* in nicotine degradation. Front Microbiol.

[CR33] Ye JB, Zhang Z, Yan J, Hao H, Liu XZ, Yang ZC, Ma K, Yang XP, Mao DP, Zhou H (2017). Degradation of phytosterols in tobacco waste extract by a novel *Paenibacillus* sp.. Biotechnol Appl Biochem.

[CR34] Yu H, Tang HZ, Wang LJ, Yao YX, Wu G, Xu P (2011). Complete genome sequence of the nicotine-degrading *Pseudomonas putida* strain S16. J Bacteriol.

[CR35] Zhang ZL, Mei XT, He ZL, Xie XY, Yang Y, Mei CY, Xue D, Hu T, Shu M, Zhong WH (2022). Nicotine metabolism pathway in bacteria: mechanism, modification, and application. Appl Microbiol Biotechnol.

[CR36] Zhao L, Zhu C, Gao Y, Wang C, Li X, Shu M, Shi Y, Zhong WH (2012). Nicotine degradation enhancement by *Pseudomonas stutzeri* ZCJ during aging process of tobacco leaves. World J Microbiol Biotechnol.

[CR37] Zhong WH, Zhu CJ, Shu M, Sun KD, Zhao L, Wang C, Ye ZJ, Chen JM (2010). Degradation of nicotine in tobacco waste extract by newly isolated *Pseudomonas* sp.. ZUTSKD Bioresour Technol.

[CR38] Zou XD, Bk A, Abu-Izneid T, Aziz A, Devnath P, Rauf A, Mitra S, Emran TB, Mujawah AAH, Lorenzo JM, Mubarak MS, Wilairatana P, Suleria HAR (2021). Current advances of functional phytochemicals in *Nicotiana* plant and related potential value of tobacco processing waste: a review. Biomed Pharmacother.

