# Supplementary material for: Cultivation and application of nicotine-degrading bacteria and environmental functioning in tobacco planting soil
Source: Bioresour Bioprocess. 2023 Feb 1;10(1):10. doi: 10.1186/s40643-023-00630-x (PMC10992035; doi:10.1186/s40643-023-00630-x)
Supplement: Supplementary file 1 — Additional file 1: Fig. S1. Nicotine metabolic pathway of bacterium Arthrobacter nitrophenolicus ND6 showing in the red box at the lower left corner. Fig. S2. Nicotine metabolic pathway of bacterium Stenotrophomonas geniculata ND16 showing in the red box at the lower left corner. Fig. S3. Color change showing by NDB under different temperatures (A) and nicotine concentrations (B). Table S1. The genomic average nucleotide identity (ANI) and digital DNA–DNA hybridization (dDDH) values between Arthrobacter sp. ND6 with affiliated species. Table S2. The genomic average nucleotide identity (ANI) and digital DNA–DNA hybridization (dDDH) values between Stenotrophomonas sp. ND16 with affiliated species. [file 40643_2023_630_MOESM1_ESM.pdf]

Additional file

Cultivation and application of nicotine-degrading bacteria and environmental functioning in tobacco planting soil

Yiting Wang[1a], Xiangyan Luo[1a], Peng Chu[1], Heli Shi[2], Rui Wang[2], Jiale Li[1], Shixue Zheng[1*]

[1] State Key Laboratory of Agricultural Microbiology, College of Life Science and Technology, Huazhong Agricultural University, Wuhan, 430070, People's Republic of China

[2] Enshi Branch, Hubei Tobacco Company, Enshi 445000, Hubei, People's Republic of China

[a] These authors contributed equally to this work.

* Corresponding author

Shixue Zheng (zhengsx@mail.hzau.edu.cn). State Key Laboratory of Agricultural Microbiology, Huazhong Agricultural University, Wuhan 430070, China. Phone: +86-27-87280670; Fax: +86-27-87280670

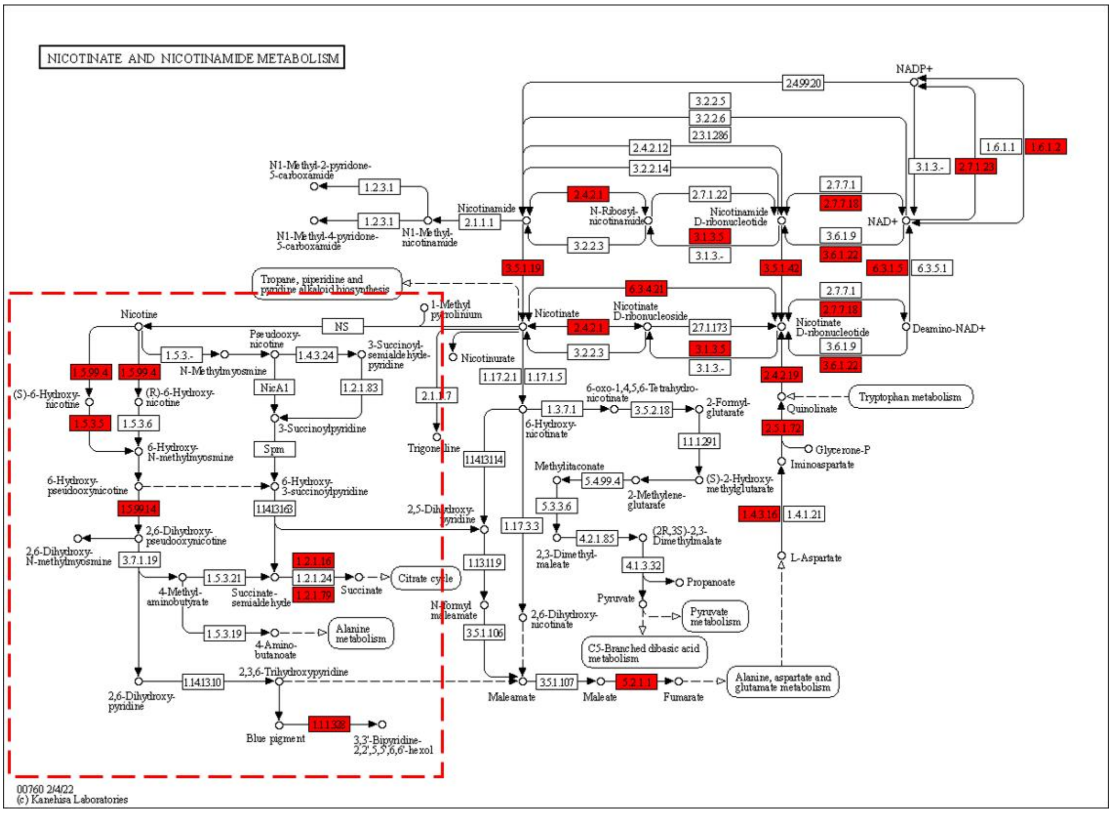

Fig. S1. Nicotine metabolic pathway of bacterium *Arthrobacter nitrophenolicus* ND6 showing in the red box at the lower left corner.

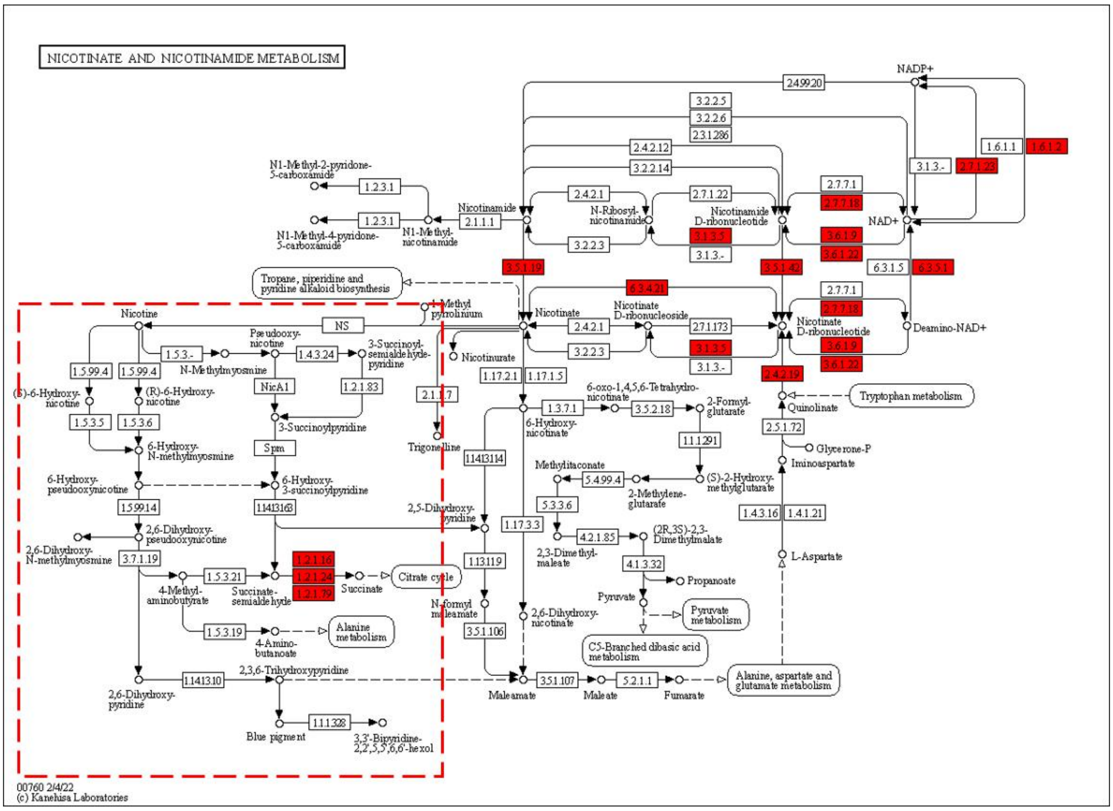

Fig. S2. Nicotine metabolic pathway of bacterium *Stenotrophomonas geniculata* ND16 showing in the red box at the lower left corner.

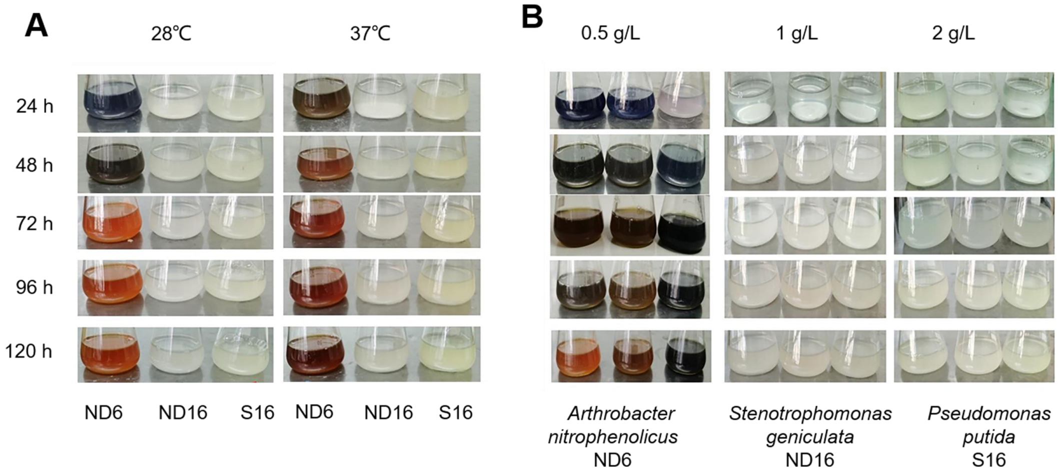

Fig. S3. Color change showing by NDB under different temperatures (A) and nicotine concentrations (B).

Table S1 The genomic average nucleotide identity (ANI) and digital DNA-DNA hybridization (dDDH) values between *Arthrobacter* sp. ND6 with affiliated species

Strains compared to *Arthrobacter* sp. ND6	Average nucleotide identity（%）	DNA–DNA hybridization values（%）
A. nitrophenolicus SJConT	97.43	86.2
A. nitrophenolicus DSM 23165	97.4	86.3
A. globiformis NBRC 12137	78.23	23.4
A. ipsi IA7	/	23.9
A. pascens DSM 20545	/	24.4
Pseudarthrobacter polychromogenes CGMCC 1.1927	81.13	42.0
P. scleromae CGMCC 1.3601	81.13	43.2
P. phenanthrenivorans Sphe3	81.42	42.8
P. siccitolerans 4J27	81.31	39.4
P. chlorophenolicus A6	80.90	37.8
P. enclensis NIO-1008	81.19	42.4
P. enclensis DSM 25279	/	42.3

*The ANI and DDH value higher than the cut-off value 95–96% and 70%, respectively proposed for the same species.

Table S2 The genomic average nucleotide identity (ANI) and digital DNA-DNA hybridization (dDDH) values between *Stenotrophomonas* sp. ND16 with affiliated species

Strains compared to *Stenotrophomonas* sp. ND16	Average nucleotide identity（%）	DNA–DNA hybridization values（%）
S. geniculata ATCC 19374	97.83	83.0
S. hibiscicola ATCC 19867	93.28	84.9
S. maltophilia NBRC 14161	92.56	74.2
S. africana LMG 22072	92.58	80.3
S. sepilia SM16975 T	90.75	73.1
S. pavanii DSM 25135	90.94	78.8
S. Maltophilia LMG 978	90.43	75.3
S. cyclobalanopsidis TPQG1-4	86.95	60.0
S.lactitubi M15	87.00	67.2
S. indicatrix WS40	86.40	71.1

* The ANI and DDH value higher than the cut-off value 95–96% and 70%, respectively proposed for the same species.
